# BioLAMR: A Biomimetically Inspired Large Language Model Adaptation Framework for Automatic Modulation Recognition

**DOI:** 10.3390/biomimetics11040288

**Published:** 2026-04-21

**Authors:** Yubo Mao, Wei Xu, Jijia Sang, Haoan Liu

**Affiliations:** 1 China Academy of Information and Communication Technology, Beijing 100191, China; maoyubo@caict.ac.cn (Y.M.); xuwei@caict.ac.cn (W.X.); 2 School of Engineering and Design, Technical University of Munich, 80333 Munich, Germany; sam.jijia@tum.de; 3 University of Chinese Academy of Sciences, Beijing 100049, China

**Keywords:** automatic modulation recognition, signal processing, large language model, communication

## Abstract

Automatic modulation recognition (AMR) is increasingly relevant to communication-sensing front ends in robotic and human–robot collaborative systems, where reliable spectrum awareness and adaptive wireless reception are desired. However, existing methods often degrade sharply at low signal-to-noise ratios (SNRs), and large language models (LLMs) are not natively compatible with continuous I/Q signals due to the inherent modality gap. We propose BioLAMR, a GPT-2 adaptation framework for AMR inspired by the auditory system’s parallel time–frequency processing and cortical hierarchy. The framework combines bio-inspired dual-domain feature extraction with parameter-efficient LLM adaptation. BioLAMR includes three components. First, a lightweight dual-domain fusion (LDDF) module extracts complementary time- and frequency-domain features and fuses them through channel and spatial attention. Second, a convolutional embedding module converts continuous I/Q signals into GPT-2-compatible sequences without discrete tokenization. Third, a hierarchical fine-tuning strategy updates only 8.9% of parameters to preserve pretrained knowledge while adapting to modulation recognition. Experiments on the RadioML2016.10a and RadioML2016.10b benchmarks show that BioLAMR achieves overall accuracies of 64.99% and 67.43%, outperforming the strongest competing method by 2.60 and 2.47 percentage points, respectively. Under low-SNR conditions, it reaches 36.78% and 38.14%, the best results among the compared methods. Ablation studies verify the contribution of each component. These results demonstrate that combining dual-domain signal modeling with parameter-efficient GPT-2 adaptation is an effective route to robust AMR in challenging wireless environments.

## 1. Introduction

With the rapid development of cognitive radio, spectrum monitoring, and intelligent communication systems, automatic modulation recognition (AMR) has become a foundational technology for efficient spectrum utilization and secure communications [1,2,3,4,5]. In non-cooperative communication scenarios, AMR enables receivers to automatically identify the modulation type of an unknown signal (e.g., AM, FM, or QPSK) without prior channel information. This capability supports subsequent signal demodulation, information extraction, and interference suppression [6,7,8,9].

In particular, the proliferation of collaborative robots, teleoperation platforms, wearable interactive devices, and unmanned systems is making wireless link awareness increasingly important in robotic and human–robot collaborative systems [10,11,12]. Robots and edge terminals in these settings often operate under protocol coexistence, severe interference, and time-varying channels. When multiple wireless standards such as Wi-Fi, Bluetooth, Zigbee, and 5G NR share the same space, timely modulation identification can support subsequent demodulation, link switching, and spectrum coexistence management [13]. Therefore, AMR can potentially serve as a front-end sensing component for robotic communication rather than only an isolated signal-processing algorithm. From a biomimetics perspective, this motivation is meaningful because the field emphasizes robust and adaptive engineering solutions inspired by biological sensing and information processing [14,15,16].

However, in practical applications, complex channel conditions (e.g., multipath fading and Doppler shift), time-varying signal characteristics, and low signal-to-noise ratio (SNR) conditions place higher demands on the generalization and feature-learning capabilities of recognition models, posing severe challenges to traditional AMR approaches. Traditional methods rely heavily on manual feature engineering, requiring domain experts to design discriminative handcrafted descriptors such as higher-order statistics and cyclostationary spectral density [17,18]. Although these methods can achieve acceptable performance in simple settings (e.g., high SNR and fixed modulation types), they adapt poorly to complex channel variations and struggle to handle increasingly diverse modulation schemes such as high-order QAM, OFDM, and APSK.

With the rise of deep learning, data-driven methods based on convolutional neural networks (CNNs), long short-term memory networks (LSTMs), and graph neural networks (GNNs) have largely replaced traditional approaches [19,20,21,22]. These models can automatically learn feature representations directly from raw in-phase/quadrature (I/Q) signals, significantly improving recognition accuracy. Nevertheless, existing deep learning-based AMR methods still suffer from inherent limitations: CNNs have limited ability to capture long-range dependencies in signal sequences; LSTMs are prone to vanishing gradients when processing long sequences; and most models are designed for fixed signal lengths, leading to limited generalization across different SNR levels and complex channel scenarios [23,24,25].

In recent years, large language models (LLMs), represented by the GPT family, have demonstrated strong capabilities in long-sequence modeling and transferable representation learning owing to self-attention mechanisms and pretraining priors [26,27,28]. Emerging studies have extended this paradigm to wireless communication tasks such as channel prediction [29] and symbol detection [30], suggesting that LLMs may be effective for modeling complex temporal correlations in signals. However, for AMR, continuous I/Q signals differ substantially from discrete text tokens in both data form and statistical structure, so directly reusing native language-model interfaces often causes feature mismatch.

From a biomimetics perspective, such cross-domain transfer resembles how biological systems preserve general perceptual priors while adapting to new tasks. More specifically, three aspects of auditory processing—parallel time–frequency analysis, cortical hierarchical processing, and selective attention—provide actionable inspiration for wireless signal-sensing module design. The guiding principle is that a model should preserve generic sequence-modeling capability while strengthening its representation of physical signal structure through lightweight task-adaptation mechanisms.

However, directly applying LLMs to AMR still faces a fundamental challenge—the modality gap. Radio signals exist as continuous I/Q dual-channel sequences with strong spatiotemporal correlations, whereas text consists of discrete token sequences organized by semantic logic. This mismatch leads to inefficient feature extraction from raw signals and prevents LLMs from fully exploiting their sequence-modeling advantages.

Motivated by these observations, this paper proposes BioLAMR—a biomimetically inspired large language model adaptation framework for AMR, with the long-term goal of supporting communication-sensing in robotics and human–robot collaboration. Rather than attempting to unify high-level interaction decision making within a single model, BioLAMR focuses on the wireless communication-sensing front end, transferring the sequence-modeling capability of GPT-2 Small to AMR through dedicated signal embedding, dual-domain feature extraction, and hierarchical fine-tuning mechanisms.

Specifically, BioLAMR comprises three core technical components. First, a lightweight dual-domain fusion module, inspired by the auditory system’s parallel time–frequency processing [31,32], extracts complementary time- and frequency-domain features in parallel and adaptively fuses them through dual-path channel and spatial attention [33]. Second, a convolutional signal embedding mechanism converts continuous I/Q signals into sequence representations compatible with LLMs. Third, a hierarchical parameter fine-tuning strategy, inspired by auditory cortical hierarchical processing [34], enables efficient task adaptation with low training cost.

The main contributions of this paper are summarized as follows:

- We propose BioLAMR, a biomimetically inspired AMR framework that adopts GPT-2 Small as the backbone and bridges continuous wireless signals and large language models through lightweight modality-adaptation mechanisms. The framework is motivated by the prospective need for robust modulation recognition in robotic and human–robot collaborative communication scenarios.
- We design a lightweight dual-domain fusion (LDDF) module that combines parallel time-domain and frequency-domain feature extraction with dual-path channel and spatial attention, thereby adaptively integrating complementary cross-domain information and enhancing feature discriminability under complex channel conditions. The module is inspired by the parallel time–frequency processing of biological auditory systems.
- We develop a convolutional signal embedding mechanism together with a hierarchical parameter fine-tuning strategy. The former bridges the modality gap between continuous signals and large language models, whereas the latter balances pretrained-knowledge retention and task-specific learning. Extensive experiments on the RadioML2016.10a and RadioML2016.10b benchmarks verify the effectiveness of the framework and provide a reusable design reference for future wireless-sensing applications.

We note that the present study validates BioLAMR exclusively on public benchmark datasets (RadioML2016.10a and RadioML2016.10b). The core contribution is therefore an algorithm-level AMR framework. The robotic and human–robot collaborative communication context is the application motivation and long-term target; actual system-level deployment and closed-loop validation on robotic platforms remain future work (discussed in Section 5).

The remainder of this paper is organized as follows: Section 2 reviews related work. Section 3 details the architecture of BioLAMR, including dual-domain fusion, signal embedding, and model fine-tuning strategies. Section 4 presents the experimental setup and analyzes the results. Section 5 discusses future directions for robotic and human–robot collaborative communications. Section 6 concludes the paper.

## 2. Related Work

This section reviews the literature from four perspectives: traditional recognition methods, deep learning-based recognition methods, biomimetic signal processing and communication studies in robotics and human–robot collaboration, and applications of large language models in wireless communications. The review clarifies the evolution of AMR, the theoretical basis of biomimetic design, and the key gaps addressed by BioLAMR.

### 2.1. Traditional Automatic Modulation Recognition Methods

Classical modulation recognition methods generally fall into two categories: likelihood-based (LB) and feature-based (FB) approaches. LB methods rely on hypothesis-testing theory to achieve Bayesian optimality using prior channel knowledge. Depending on how parameter uncertainty is handled, they can be divided into average, generalized, and hybrid likelihood ratio tests (ALRT, GLRT, and HLRT) [35,36]. Although theoretically rigorous, LB methods suffer from high computational complexity and sensitivity to unknown parameters, which limits their applicability in real-time scenarios.

By contrast, FB methods sacrifice some accuracy for lower complexity, making them more suitable for real-time applications. These approaches extract handcrafted features such as instantaneous time-domain statistics [37,38], higher-order cumulants [39], and transform-domain patterns [40], followed by classification with algorithms such as support vector machines (SVMs) [41]. Time-domain features are computationally efficient but sensitive to fading; higher-order cumulants are more noise-resilient but struggle to distinguish modulations with similar statistics; and transform-domain features capture multiscale details but depend heavily on expert knowledge. Fundamentally, both paradigms rely on manual feature engineering and prior assumptions, which restrict their generalization to challenging low-SNR conditions and emerging modulation schemes.

### 2.2. Deep Learning-Based Automatic Modulation Recognition Methods

With advances in automatic feature extraction and sequence modeling, deep learning-based AMR has become the dominant paradigm. These models learn hierarchical representations directly from raw I/Q signals and substantially reduce reliance on manual feature design.

Representative studies span several model families. Lee et al. [42] extracted 28 handcrafted features under fading conditions and trained a four-layer DNN to recognize multiple modulations. CNN-based methods excel at capturing spatial features and local patterns; O’Shea et al. [43] proposed the VTCNN baseline and released the RadioML2016.10a benchmark. RNN-based methods, such as attention-enhanced LSTMs [44], focus on temporal dependencies. Hybrid CNN–RNN architectures such as CLDNN [45] combine convolutional feature extraction with LSTM-based temporal modeling. However, deep learning methods still face several common challenges: feature discriminability declines markedly under low SNR, large-scale labeled data are needed despite the high cost of annotation, and increasing model complexity does not always yield proportional gains. For example, even complex architectures such as deep transformers [46] still exhibit clear accuracy bottlenecks under low-SNR conditions. More recently, Lei et al. [47] proposed a fully complex-valued transformer for modulation recognition, in which the received signals are processed in the complex domain to preserve the intrinsic relationship between the real and imaginary components; both theoretical analysis and experiments showed advantages over real-valued counterparts and other benchmarks.

### 2.3. Biomimetic Signal Processing and Communications in Robotics and Human–Robot Collaboration

Biomimetics addresses engineering problems by imitating biological structures and functional principles, and it has profoundly influenced signal processing and intelligent communications [48,49,50]. At the signal-sensing level, the biological auditory system provides direct inspiration for multi-domain analysis: the mammalian cochlea performs real-time frequency decomposition through the mechanical filtering of the basilar membrane, while hair cells preserve fine temporal transient responses [31,32]. This time–frequency parallel biological architecture has inspired a range of bio-inspired signal processing methods, including Gammatone filter banks [51], which have been widely used in speech recognition and acoustic scene classification. In addition, the application potential of biomimetic tactile microstructures in texture recognition, health monitoring, and human–machine interaction further indicates that bio-inspired multimodal perception mechanisms are becoming an important foundation for intelligent interactive systems [52]. However, little work has investigated how such bio-inspired time–frequency parallel processing ideas can be transferred to modulation recognition for radio signals.

In robotic and human–robot collaborative communications, the growing adoption of collaborative robots [10], teleoperation systems [53], and wearable intelligent devices [15,32,54] has made robust communication-link sensing critical to interaction quality and system safety. In complex electromagnetic environments such as factory floors, disaster response, and medical assistance, the coexistence of multiple wireless protocols places higher demands on the receiver’s ability to interpret signals [55]. Bio-inspired perception and indoor navigation mechanisms for service robots, as well as AI-driven biomimetic robot-control strategies, indicate that robotic systems operating in complex environments increasingly rely on perception modules that are both robust and adaptive [14,16]. Nevertheless, most existing AMR studies focus primarily on communication performance itself, with limited discussion of real-time operation, robustness, and resource constraints from the standpoint of robotic communication-sensing front ends.

### 2.4. Exploration of Large Language Model Applications in Wireless Communications

In recent years, large language models have shifted wireless communication research from task-specific modeling toward more general representation-learning frameworks [56,57,58]. These models are particularly attractive because of their strong long-sequence modeling ability, cross-task transferability, and context-aware representation capabilities.

Current studies have expanded in several main directions. In semantic communications, LLMs act as shared knowledge bases for semantic source coding, extracting and reconstructing meaning beyond conventional transmission metrics to improve communication efficiency [59,60]. In network optimization, LLMs exploit their reasoning capability to realize intent-driven networking, automatically translating high-level user requirements into network-configuration parameters [61]. In addition, to cope with increasingly complex 3GPP standards, LLMs have been adapted as intelligent question-answering assistants by combining retrieval-augmented generation and domain-specific fine-tuning, thereby supporting tasks ranging from network troubleshooting to software development [62].

However, the application of LLMs to AMR remains in its early stages, and the core bottlenecks are still the modality gap and insufficient task adaptation. On the one hand, radio signals are continuous I/Q dual-channel sequences with strong spatiotemporal correlations and explicit physical structure, whereas language models are inherently designed for discrete token sequences. On the other hand, modulation recognition requires simultaneous modeling of local waveforms and global spectral information, yet mature LLM adaptation frameworks for this setting are still lacking. Therefore, designing efficient adaptation mechanisms to bridge LLMs and AMR tasks remains a key research direction and is the main entry point of this work.

## 3. Proposed Method

**Figure 1:**
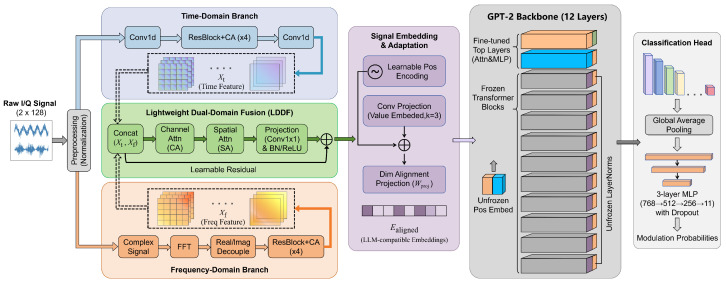
This flowchart illustrates the overall architecture and core modules of BioLAMR, showing the complete pipeline from input time-series signals to output modulation-recognition results.

**Figure 2:**
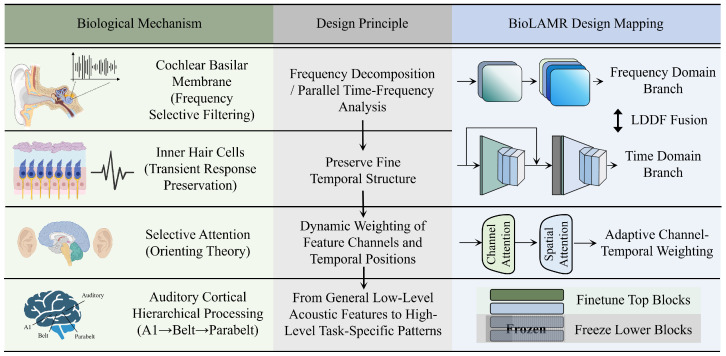
Mapping from biological inspirations to design principles and corresponding modules in BioLAMR. The figure highlights how auditory time–frequency analysis, selective attention, and cortical hierarchical processing motivate the dual-domain branches, the LDDF module, and the hierarchical fine-tuning strategy, respectively.

This section details the architecture and core components of the BioLAMR framework. We adopt pretrained GPT-2 Small as the backbone because it models long-range temporal dependencies with a moderate computational footprint. Inspired by biological perceptual systems, the framework has three objectives. It exploits complementary time–frequency information through dual-domain feature extraction, bridges the modality gap between continuous radio signals and the pretrained backbone through convolutional signal embedding, and balances pretrained-knowledge reuse with task adaptation through hierarchical parameter fine-tuning. Figure 1 illustrates the overall architecture of the framework, while Figure 2 summarizes the biomimetic inspirations, and the resulting design principles in BioLAMR.

### 3.1. Overall Architecture

BioLAMR adopts an end-to-end trainable architecture. Given an input I/Q signal sample $x\in R^{C\times L}$, where C=2 denotes the I/Q dual channels and *L* is the sequence length, the forward process proceeds as follows. The signal is first per-sample amplitude-normalized to remove scale variations introduced by channel impairments. It is then processed by the dual-domain feature extraction module, which models local transient characteristics and spectral distributions in parallel. The time-domain and frequency-domain features are fused through an adaptive fusion mechanism. The fused representation is converted by the signal embedding layer into a high-dimensional sequence compatible with GPT-2. Finally, the GPT-2 backbone models the global dependencies of the embedded sequence, and the pooled output is fed to the classification head for modulation prediction.

The central design philosophy of BioLAMR is to balance knowledge reuse and task adaptation. On the one hand, most GPT-2 parameters are kept frozen, and only key layers such as LayerNorm, position embeddings, and top-layer attention blocks are updated, thereby maximizing reuse of sequence-modeling priors acquired from large-scale text pretraining. On the other hand, lightweight adaptation modules tailored to radio signals—namely dual-domain fusion and signal embedding—are introduced to bridge the modality gap between continuous I/Q waveforms and discrete token representations with minimal additional parameters. This design enables effective transfer of pretrained knowledge to AMR while maintaining relatively low computational overhead.

### 3.2. Dual-Domain Feature Extraction

Discriminative features of modulated signals exist in both the time and frequency domains: time-domain waveforms reflect transient characteristics such as symbol boundaries and phase transitions, whereas frequency-domain spectral morphology encodes global patterns such as spectral envelopes and energy distribution. This property is closely analogous to the operation of mammalian auditory systems, in which the basilar membrane performs frequency decomposition while inner hair cells (IHCs) preserve fine temporal transients [31,32]. The parallel dual-branch design is therefore not purely metaphorical. Biological auditory systems use parallel time–frequency processing to resolve a fundamental information-theoretic trade-off: temporal resolution and frequency resolution are inversely coupled. Radio signals face a similar constraint. Time-domain waveforms preserve transient phase details but provide limited global spectral context, whereas frequency-domain representations capture spectral envelopes but sacrifice instantaneous phase information. A parallel architecture is therefore a practical way to address the same computational bottleneck. Inspired by this biologically motivated observation, we design parallel feature-extraction branches with symmetric residual architectures to extract complementary information from both domains.

A natural alternative for joint time–frequency analysis is the short-time Fourier transform (STFT). However, STFT is constrained by the Heisenberg uncertainty principle: once the window length is fixed, improving time resolution degrades frequency resolution, and vice versa. For the short signal segments used in this study (L=128), any practical STFT window leaves limited resolution in at least one domain. The parallel dual-branch design therefore avoids compressing both cues into a single fixed-window representation. The time branch keeps the full *L*-point temporal resolution, whereas the frequency branch keeps the full *L*-point spectral resolution. In addition, the frequency branch retains both real and imaginary DFT components through Cartesian decomposition, which preserves phase information important for discriminating phase-shift keying signals such as BPSK, QPSK, and 8PSK. A complex-valued STFT could also retain phase, but common magnitude- or log-magnitude spectrogram pipelines discard this cue. Retaining the full complex STFT would also produce a higher-dimensional time–frequency tensor and require a substantially different encoder. Finally, both branches output one-dimensional sequences ($R^{C\times L}$) that are directly compatible with the downstream transformer backbone, whereas an STFT representation would require either a 2D encoder or a nontrivial reshaping step before entering GPT-2.

Notation. As shown in Table 1, we first establish the mathematical notation used throughout this subsection. Let B={1,2,…,B} denote the batch index set, and define the following key variables:

For any tensor $T\in R^{d_{1}\times d_{2}\times \ldots \times d_{n}}$, we denote by T[i,:,…,:] the *i*-th slice along the first dimension. The Hadamard product ⊗ is defined element-wise: ${\left(A⊗B\right)}_{ij}=A_{ij}\cdot B_{ij}$.

Branch 1: Time-Domain Processing

The time-domain branch defines a mapping $F_{\mathrm{time}}:R^{C\times L}\to R^{C\times L}$ that extracts local structural features from raw I/Q signals through a shallow convolutional residual network. Given the normalized signal $x_{\mathrm{norm}}\in R^{C\times L}$ (where C=2), the processing flow consists of three steps:

Initial Feature Expansion: Define the initial convolutional operator $\phi_{0}:R^{C\times L}\to R^{d_{\mathrm{res}}\times L}$ as:

(1) $$F_{t}^{\left(0\right)}=\phi_{0}\left(x_{\mathrm{norm}}\right)={\mathrm{Conv}1d}^{k=3}\left(x_{\mathrm{norm}};\theta_{0}\right)$$

where $\theta_{0}\in R^{d_{\mathrm{res}}\times C\times 3}$ represents the learnable kernel parameters, and $d_{\mathrm{res}}\in N$ denotes the intermediate feature dimension. This establishes the mapping $R^{C\times L}\to R^{d_{\mathrm{res}}\times L}$.

Hierarchical Residual Learning: For k∈{1,2,…,K}, define the residual block operator $R_{k}:R^{d_{\mathrm{res}}\times L}\to R^{d_{\mathrm{res}}\times L}$ with identity skip connection:

(2) $$F_{t}^{\left(k\right)}=F_{t}^{\left(k-1\right)}+G_{k}\left(F_{t}^{\left(k-1\right)}\right)$$

where the residual function $G_{k}$ is composed as:

(3) $$G_{k}=\mathrm{CA}\circ \phi_{k,2}\circ \mathrm{ReLU}\circ \phi_{k,1}$$

Here, $\phi_{k,1},\phi_{k,2}$ denote convolutional operators, and CA represents the channel attention mechanism.

The channel attention operator $\mathrm{CA}:R^{d\times L}\to R^{d\times L}$ is defined via spatial pooling followed by channel-wise weight generation:

(4) $$\begin{array}{c} \mathrm{CA}\left(F\right)=\left(\sigma (\psi (GAP(F)))\right)⊗F \end{array}$$

(5) $$\begin{array}{c} \mathrm{where}\;GAP\left(F\right)=\frac{1}{L}\sum_{l=1}^{L}F\left[:,l\right] \end{array}$$

and $\psi :R^{d}\to R^{d}$ is an MLP mapping (with bottleneck dimension d/r, where *r* is the reduction ratio). The operator σ:R→(0,1) denotes the sigmoid function applied element-wise, and ⊗ denotes the Hadamard product with broadcasting.

Dimension Recovery: Finally, project the features back to the original channel space via $\phi_{\mathrm{out}}:R^{d_{\mathrm{res}}\times L}\to R^{C\times L}$:

(6) $$X_{t}=\phi_{\mathrm{out}}\left(F_{t}^{\left(K\right)}\right)={\mathrm{Conv}1d}^{k=3}\left(F_{t}^{\left(K\right)};\theta_{\mathrm{out}}\right)$$

The complete time-domain mapping is therefore: $X_{t}=F_{\mathrm{time}}\left(x_{\mathrm{norm}}\right)=\left(\phi_{\mathrm{out}}\circ R_{K}\circ \cdots \circ R_{1}\circ \phi_{0}\right)\left(x_{\mathrm{norm}}\right)$.

This design balances expressive power and parameter scale through a shallow architecture (totaling approximately $2K\times d_{\mathrm{res}}^{2}$ parameters), thereby reducing the risk of overfitting. More specifically, the robustness to noise arises from adaptive feature selection rather than from a dedicated denoising stage. First, the stacked local convolutions (kernel size k=3) tend to reinforce waveform structures that persist across adjacent samples—such as amplitude transitions and envelope contours—while temporally uncorrelated additive perturbations are less likely to be consistently reinforced through successive layers. Second, the identity skip connections in Equation (2) preserve coarse signal structure: when the residual branch $G_{k}$ contributes little useful refinement under heavy noise, the block output remains close to its input, reducing the risk that noisy fluctuations are progressively amplified across depth. Third, the channel-attention operator in Equation (4) aggregates each feature channel over the full sequence via global average pooling and generates channel-wise sigmoid gates, so channels that respond consistently to modulation-dependent patterns receive higher weights, whereas channels that are less consistently aligned with such patterns can receive lower weights. Together, these three mechanisms enable the time-domain branch to emphasize discriminative signal patterns while limiting noise propagation through the network.

Branch 2: Frequency-Domain Processing

The frequency-domain branch defines a composite mapping $F_{\mathrm{freq}}:R^{C\times L}\to R^{C\times L}$ that captures global spectral characteristics via Fourier analysis. This mapping decomposes into three cascaded stages:

Complex Signal Formation: Define the complexification operator $C:R^{2\times L}\to C^{L}$ as:

(7) $$z=C\left(x_{\mathrm{norm}}\right)=x_{\mathrm{norm}}\left[0,:\right]+j\cdot x_{\mathrm{norm}}\left[1,:\right]$$

where $j=\sqrt{-1}$ denotes the imaginary unit, and $x_{\mathrm{norm}}\left[i,:\right]$ extracts the *i*-th channel (i∈{0,1} for I/Q components).

Fourier Transformation: Apply the discrete Fourier transform $F:C^{L}\to C^{L}$, whose *k*-th coefficient is defined by:

(8) $$Z[k]=\sum_{l=0}^{L-1}z\left[l\right]\cdot e^{-2\pi j\cdot kl/L},\;k\in \left\{0,1,\ldots ,L-1\right\}$$

where $Z=F\left(z\right)\in C^{L}$ denotes the spectral coefficient vector. The spectral coefficients encode the frequency-domain representation of the input signal, satisfying Parseval’s theorem: ${∥z∥}_{2}^{2}=\frac{1}{L}{∥Z∥}_{2}^{2}$.

Real–Imaginary Decoupling: Define the Cartesian decomposition operator $D:C^{L}\to R^{2\times L}$ as:

(9) xfreq=D(Z)=Re(Z)Im(Z)

where Re,Im:CL→RL extract real and imaginary components, respectively.

Spectral Feature Extraction: The decoupled frequency-domain signal $x_{\mathrm{freq}}\in R^{C\times L}$ is processed by a convolutional residual network $G_{\mathrm{freq}}:R^{C\times L}\to R^{C\times L}$ that is structurally isomorphic to the time-domain branch:

(10) $$X_{f}=G_{\mathrm{freq}}\left(x_{\mathrm{freq}}\right)$$

The complete frequency-domain mapping is the composition: $X_{f}=F_{\mathrm{freq}}\left(x_{\mathrm{norm}}\right)=\left(G_{\mathrm{freq}}\circ D\circ F\circ C\right)\left(x_{\mathrm{norm}}\right)$.

By transforming the signal with an FFT, the model can access global statistical characteristics of modulation, such as spectral morphology and energy concentration, beyond local waveform details. From a signal-processing perspective, frequency-domain features are generally more stable than raw waveforms under impairments such as multipath fading and small frequency offsets, because the channel’s impulse response appears as smooth spectral shaping, to which spectral representations are comparatively robust.

### 3.3. Lightweight Dual-Domain Fusion

**Figure 3:**
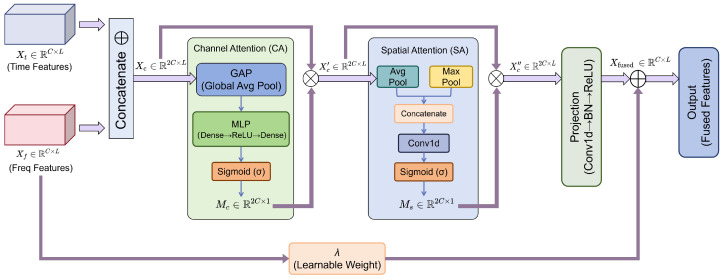
Schematic diagram of the lightweight dual-domain fusion (LDDF) mechanism, illustrating the flow from dual-domain inputs to the fused feature output. CA and SA denote channel attention and spatial attention, respectively.

The fusion of time–frequency information is handled by a learnable lightweight dual-domain fusion (LDDF) module. Its attention-driven design is inspired by selective-attention mechanisms in cognitive neuroscience [33]: channel attention corresponds to feature-based attention, which allocates processing resources across different feature channels, whereas spatial attention corresponds to position-based attention along the time axis. This dual-pathway separation is grounded in a specific functional hypothesis: information selection is most efficient when what(feature identity) and where (temporal position) are handled by complementary, interacting pathways rather than a single monolithic mechanism [33]. For modulated signals, channel attention learns to weight the relative importance of time-domain versus frequency-domain features per sample, while spatial attention highlights temporally informative positions such as symbol boundaries and phase transitions. This structured separation of concerns is therefore expected to be more effective than unweighted concatenation or a single-path attention mechanism. Specifically, with $X_{t}$ and $X_{f}$ (both in $R^{C\times L}$, with C=2) as inputs, the module uses dual-path channel and spatial attention to capture complementary features and outputs a fused tensor with the same dimensions as the inputs. Figure 3 provides a schematic overview of its five-stage lightweight architecture.

Stage 1: Feature Concatenation

Define the channel-wise concatenation operator $⊕:R^{C\times L}+R^{C\times L}\to R^{2C\times L}$ as:

(11) $$X_{c}=X_{t}⊕X_{f}=\left[\begin{array}{c} X_{t}\\ X_{f} \end{array}\right]$$

This operation constructs the joint feature space $R^{2C\times L}$, where the entry $X_{c}\left[i,l\right]$ at temporal indices *l* corresponds to time-domain features for i≤C and frequency-domain features for i>C.

Stage 2: Channel Attention Mechanism

Define the channel attention operator $M_{c}:R^{2C\times L}\to R^{2C\times L}$ which learns an adaptive weighting over feature channels. This consists of three sub-operations: The LDDF channel-attention block adopts a lightweight gating formulation, similar in spirit to channel-attention modules such as CBAM [63]. The concatenated feature map is first compressed into a global descriptor $D_{c}$, which is then mapped to channel gates $M_{c}$ through a bottleneck MLP and sigmoid activation; the resulting gates rescale each channel of $X_{c}$ multiplicatively.

Spatial Aggregation: Define the global average pooling operator $P_{\mathrm{spatial}}:R^{2C\times L}\to R^{2C}$ as:

(12) $$D_{c}=P_{\mathrm{spatial}}\left(X_{c}\right)=\frac{1}{L}\sum_{l=1}^{L}X_{c}\left[:,l\right]$$

This operation aggregates the feature map into global context descriptors.

Channel Weight Generation: Define a two-layer MLP $\Phi_{c}:R^{2C}\to R^{2C}$ with bottleneck architecture:

(13) $$\Phi_{c}\left(D_{c}\right)=W_{1}\left(\mathrm{ReLU}\left(W_{0}\left(D_{c}\right)\right)\right)$$

where $W_{0}\in R^{C\times 2C}$ and $W_{1}\in R^{2C\times C}$ are learnable weight matrices implementing dimension reduction and restoration. Specifically, the channel attention operator $M_{c}$ generates a channel attention map $M_{c}\in R^{2C}$:

(14) $$M_{c}=\sigma \left(\Phi_{c}\left(D_{c}\right)\right)$$

where σ:R→(0,1) is the sigmoid function applied element-wise, producing a soft gating vector $M_{c}\in {\left(0,1\right)}^{2C}$.

Channel Modulation: Apply the attention weights via the Hadamard product with broadcasting:

(15) $$X_{c}^{\prime }=M_{c}\left(X_{c}\right)=M_{c}⊗X_{c}=\left[\begin{array}{c} M_{c}\left[1\right]\cdot X_{c}\left[1,:\right]\\ \vdots \\ M_{c}\left[2C\right]\cdot X_{c}\left[2C,:\right] \end{array}\right]$$

This mechanism enables adaptive importance weighting: $M_{c}\left[i\right]\to 1^{-}$ for discriminative channels and $M_{c}\left[i\right]\to 0^{+}$ for redundant ones.

Stage 3: Spatial Attention Mechanism

Define the spatial attention operator $M_{s}:R^{2C\times L}\to R^{2C\times L}$ which learns position-wise importance weights along the temporal dimension: The spatial-attention block likewise performs position-wise saliency reweighting along the temporal axis. It first compresses the channel dimension into two summary sequences, $D_{s,\mathrm{avg}}$ and $D_{s,\max}$, then applies a local convolution to generate a scalar gate $M_{s}\left[l\right]$ for each temporal position, and finally broadcasts this gate along channels to modulate $X_{c}^{\prime }$.

Channel Aggregation: Define two complementary channel pooling operators $P_{\mathrm{avg}},P_{\max}:R^{2C\times L}\to R^{1\times L}$:

(16) $$\begin{array}{cc} D_{s,\mathrm{avg}} & =P_{\mathrm{avg}}\left(X_{c}^{\prime }\right)=\frac{1}{2C}\sum_{c=1}^{2C}X_{c}^{\prime }\left[c,:\right] \end{array}$$

(17) $$\begin{array}{cc} D_{s,\max} & =P_{\max}\left(X_{c}^{\prime }\right)=\max_{c\in \{1,\ldots ,2C\}}X_{c}^{\prime }\left[c,:\right] \end{array}$$

These capture average activation and maximum response patterns across channels.

Spatial Descriptor Fusion: Stack the two descriptors to form a bivariate spatial feature:

(18) $$S=\left[\begin{array}{c} D_{s,\mathrm{avg}}\\ D_{s,\max} \end{array}\right]$$

Spatial Weight Generation: Apply a convolutional operator $\Psi_{s}:R^{2\times L}\to R^{1\times L}$ with kernel width $k_{s}$ to capture local temporal context:

(19) $$M_{s}\left[l\right]=\sigma \left(\sum_{i=0}^{k_{s}-1}w_{i}^{⊤}S\left[:,l-\left\lfloor k_{s}/2\right\rfloor +i\right]\right),\;l\in \{1,\ldots ,L\}$$

where $w\in R^{k_{s}\times 2}$ denotes the learnable convolution kernel. Specifically, $w_{i}\in R^{2}$ denote the weight vector at the *i*-th spatial position of the kernel, which performs an inner product to fuse the bivariate spatial descriptors (Avg and Max) into a scalar intensity.

Spatial Modulation: Apply the position-wise attention via broadcasting:

(20) $$X_{c}^{\prime \prime }=M_{s}\left(X_{c}^{\prime }\right)=M_{s}⊗X_{c}^{\prime }=\left[\begin{array}{c} M_{s}⊙X_{c}^{\prime }\left[1,:\right]\\ \vdots \\ M_{s}⊙X_{c}^{\prime }\left[2C,:\right] \end{array}\right]$$

where ⊙ denotes element-wise multiplication. This produces position-adaptive feature modulation: $M_{s}\left[l\right]\to 1^{-}$ for informative temporal indices (e.g., symbol transitions) and $M_{s}\left[l\right]\to 0^{+}$ for noise-dominated regions.

Stage 4: Channel Projection and Normalization

Define the projection operator $\Pi :R^{2C\times L}\to R^{C\times L}$ that reduces dimensionality via pointwise (1×1) convolution followed by batch normalization and nonlinear activation:

(21) $$X_{\mathrm{fused}}=\mathrm{ReLU}\left(N\left(\Pi \left(X_{c}^{\prime \prime }\right)\right)\right)$$

where:

- $\Pi \left(X\right)=W_{\mathrm{proj}}\cdot X$ with $W_{\mathrm{proj}}\in R^{C\times 2C}$ being learnable projection weights.
- $N:R^{C\times L}\to R^{C\times L}$ denotes batch normalization.
- ReLU(x)=max(0,x) is the rectified linear unit.

This establishes the mapping $R^{2C\times L}\to R^{C\times L}$.

Stage 5: Learnable Residual Connection

To preserve the input manifold structure while injecting cross-domain information, define the final fusion mapping with a parameterized skip connection:

(22) $$Y=A\left(X_{t},X_{f}\right)=X_{\mathrm{fused}}+\lambda \cdot X_{t}$$

where λ∈R is a learnable scalar weight that balances between:

- Identity path: $X_{t}$ preserves the original time-domain representation.
- Fusion path: $X_{\mathrm{fused}}$ introduces frequency-domain complementarity.

(23) $$X_{\mathrm{fused}}=\mathrm{ReLU}\left(N\left(\Pi \left(M_{s}\left(M_{c}\left(X_{t}⊕X_{f}\right)\right)\right)\right)\right)$$

This composition ensures gradient flow stability via the residual path while enabling adaptive domain fusion via the attention mechanisms $M_{c}$ and $M_{s}$.

This fusion mechanism offers three advantages: (1) Adaptability: dual-path attention enables the model to dynamically adjust time- and frequency-domain weights according to sample characteristics; (2) Lightweight: the fusion module introduces minimal parameters with negligible computational overhead; and (3) Compatibility: it preserves consistent input–output dimensions (both 2×L), enabling seamless connection to subsequent embedding layers.

### 3.4. Signal Embedding and GPT-2 Adaptation

Unlike naturally discrete text tokens, I/Q signals are continuous-valued sequences and therefore require a dedicated embedding strategy to bridge the modality gap. The cross-modal adaptation from continuous I/Q signals to transformer-compatible representations is defined by an embedding function $E:R^{C\times L}\to R^{L\times d_{\mathrm{gpt}}}$ that preserves signal structure while enabling self-attention. This mapping consists of three stages.

Stage 1: Value Embedding via Convolutional Projection

Rather than treating sampling points as independent tokens, we treat the fused signal *Y* as a coherent waveform. We generate the value embedding sequence $E_{\mathrm{value}}\in R^{L\times d_{\mathrm{gpt}}}$ by applying a 1D convolutional operator with kernel size k=3:

(24) $$E_{\mathrm{value}}={\mathrm{Conv}1d}^{k=3}\left(Y;W,b\right)$$

where *W* and *b* denote learnable weights and bias. Intuitively, this operation slides a window of size 3 across the signal and aggregates information from the local neighborhood, including the previous, current, and subsequent time steps. With the same padding, the output retains the original sequence length *L*, thereby capturing smooth waveform transitions and local trends without compressing the data.

Stage 2: Learnable Position Encoding

To inject temporal ordering information, we define a position-encoding function $P\left(\cdot \right)\in R^{d_{\mathrm{gpt}}}$ that maps temporal index *l* to a dense vector. This function is parameterized by a learnable embedding matrix $P\in R^{L_{\max}\times d_{\mathrm{gpt}}}$, where $L_{\max}$ is the maximum supported sequence length. For a sequence of length *L* ($L\leq L_{\max}$), the position vector is obtained by slicing:

(25) P(l)=P[l,:],∀l∈{1,…,L}

The final embedding is then formed by adding position information to the value embedding element-wise:

(26) $$E\left[l,:\right]=E_{\mathrm{value}}\left[l,:\right]+P\left(l\right)$$

This produces a combined representation $E\in R^{L\times d_{\mathrm{gpt}}}$ that encodes both local waveform structure and global temporal position. The use of learnable positional encoding is motivated by the fact that GPT-2’s pretrained positional representations are optimized for natural-language token order and cannot be directly assumed to capture the strong physical periodicity of radio signals. In wireless signal processing, temporal position encodes meaningful periodic information such as carrier phase states and symbol boundaries rather than merely sequential order. Therefore, we introduce a newly initialized learnable matrix *P* so that BioLAMR can adaptively learn modulation-specific temporal patterns from signal data.

Stage 3: Feature Distribution Alignment and Transformer Interface

To align the feature distribution of signal embeddings with the input expectation of pretrained GPT-2 hidden states, we introduce a linear projection layer $\Pi_{\mathrm{align}}:R^{L\times d_{\mathrm{gpt}}}\to R^{L\times d_{\mathrm{gpt}}}$ defined as

(27) $$E_{\mathrm{aligned}}=\Pi_{\mathrm{align}}\left(E\right)=E\cdot W_{\mathrm{align}}^{⊤}$$

where $W_{\mathrm{align}}\in R^{d_{\mathrm{gpt}}\times d_{\mathrm{gpt}}}$ is a learnable square projection used to adapt signal-domain features to GPT-2’s input space.

At this stage, the aligned embedding vectors are seamlessly integrated into GPT-2 through its embedding interface. This design is crucial because it bypasses discrete tokenization entirely and preserves the rich continuous information carried by radio signals. Instead of forcing continuous-valued waveforms into a text-based token space—which would inevitably quantize and destroy fine-grained signal characteristics—we directly inject continuous vectors into the transformer’s processing pipeline.

Subsequently, these embeddings pass through the deep GPT-2 architecture, which acts as a hierarchical feature extraction pipeline: each transformer block uses multi-head self-attention to interrogate the signal at multiple temporal scales, followed by feed-forward networks that distill increasingly abstract representations. This multi-stage processing produces highly contextualized embeddings capable of capturing both local waveform attributes and long-range temporal dependencies, both of which are essential for identifying complex modulation patterns.

**Figure 4:**
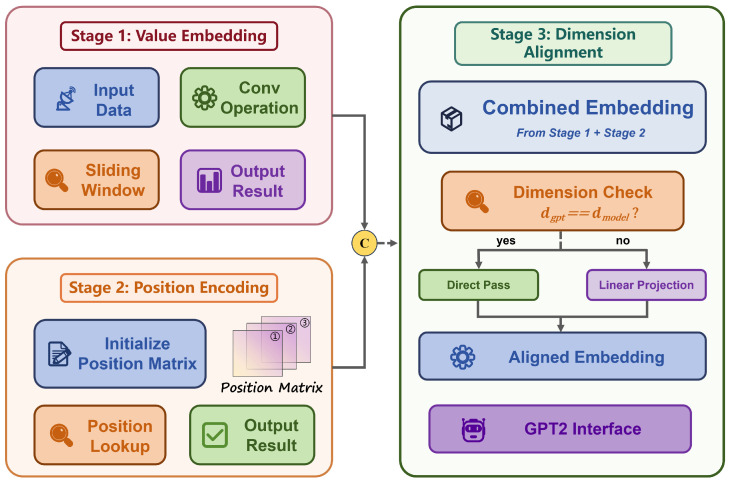
Architecture of the signal embedding and GPT-2 adaptation pipeline in BioLAMR. The input signals undergo a three-stage transformation into continuous vector representations, which are directly injected into the GPT-2 backbone while bypassing discrete tokenization to preserve signal continuity and enable end-to-end optimization.

Figure 4 depicts the overall workflow. Mathematically, the complete pipeline from the input signal to the GPT-2 hidden representation can be formalized as

(28) $$H=T_{\mathrm{GPT}-2}\circ \Pi_{\mathrm{align}}\circ \left(\Theta_{v}+P\right)$$

where the first three stages constitute the embedding function $E=\Pi_{\mathrm{align}}\circ \left(\Theta_{v}+P\right)$. This composite formulation reflects a sequential transformation process that begins with value embedding and positional encoding, proceeds to feature-distribution alignment, and ends with deep representation learning through the GPT-2 transformer backbone. The main advantage of this design is that it preserves signal continuity throughout the pipeline, avoids quantization errors associated with discrete tokenization, and enables GPT-2’s self-attention to model long-range temporal patterns that are often obscured by local noise.

### 3.5. Hierarchical Parameter Fine-Tuning Strategy

Blindly fine-tuning all GPT-2 parameters introduces two major challenges: (1) the large parameter space of GPT-2 Small (approximately 124 million parameters) substantially increases the risk of overfitting on modulation-recognition datasets, and (2) aggressive updates can cause catastrophic forgetting, eroding the generic sequence-modeling capability of the pretrained backbone. To mitigate these risks, and inspired by hierarchical processing in the auditory cortex [34], we introduce a hierarchical parameter-unfreezing strategy. Neuroscience studies on the auditory cortical hierarchy suggest that primary areas (e.g., A1) extract generic low-level acoustic features, whereas higher belt and parabelt areas become increasingly selective for complex and task-relevant patterns. Analogously, our strategy keeps lower GPT-2 layers frozen and progressively unfreezes selected top-layer parameters so as to balance prior-knowledge retention and task adaptation. Notably, the cortical-hierarchy analogy does not merely relabel a generic parameter-efficiency heuristic: rather than freezing an arbitrary subset of layers to reduce trainable parameters, it prescribes a specific unfreezing direction—bottom layers frozen for generic sequence modeling, top layers unfrozen for task specialization—mirroring the functional gradient from primary to higher-order auditory areas.

By default, all GPT-2 parameters are frozen, including self-attention modules, feed-forward networks, and token embeddings. Initially, only two groups of parameters are unfrozen:

1. LayerNorm parameters: the scaling and offset parameters (γ and β) of all transformer LayerNorm layers are updated so that the model can quickly recalibrate the distribution mismatch between I/Q signals and text data.
2. Position encoding: the learnable position matrix *P* is updated so that the model can capture temporal patterns specific to modulation signals, whose periodic structure differs fundamentally from natural language.

This initialization stage greatly reduces the risk of overfitting while maintaining efficient reuse of pretrained knowledge. After the initial adaptation of LayerNorm and positional encodings, typically over 5–10 epochs, we progressively unfreeze the top-layer parameters so that the model can learn task-specific discriminative patterns.

1. Top-layer attention: the self-attention weights of the last two transformer blocks are unfrozen. This choice balances computational efficiency and adaptation flexibility, allowing the model to learn modulation-specific global attention patterns.
2. Final MLP: the feed-forward network of the last transformer block is unfrozen so that the high-dimensional feature space can be realigned specifically for AMR, thereby improving the separability of modulation classes.

Lower transformer blocks remain frozen to preserve the stability of basic sequence modeling. This hierarchical strategy follows the principle of “general lower layers, task-specific upper layers”. Table 2 summarizes the parameter allocation and trainable status of each component in GPT-2 Small. Ultimately, only about 8.9% of the total parameters are trainable, enabling efficient task adaptation while preserving pretrained knowledge.

## 4. Experiments

This section evaluates BioLAMR through comparative experiments, ablation studies, and feature-space visualizations. The goals are to (i) compare the proposed framework against representative deep-learning baselines across different SNR regimes, (ii) isolate the contributions of dual-domain fusion, convolutional embedding, and hierarchical fine-tuning through controlled ablations, and (iii) examine how pretrained GPT-2 knowledge transfers to wireless signal processing.

### 4.1. Experimental Setup

#### 4.1.1. Dataset Description and Physical Characteristics

To ensure the reproducibility of our results and facilitate a fair comparison with the mainstream literature, this study uses two widely recognized benchmark datasets in wireless communications: RadioML2016.10a and RadioML2016.10b. Both were generated using the GNU Radio software-defined radio platform and cover an SNR range from −20 dB to +18 dB (in 2 dB steps). Each sample is a complex-valued signal vector of length L=128 with two channels (in-phase/quadrature, I/Q). However, the two datasets differ in important ways:

- RadioML2016.10a: contains 11 modulation types under relatively ideal channel conditions, with simulated center-frequency and sample-rate offsets modeled as random-walk processes.
- RadioML2016.10b: contains 10 modulation types (without AM-SSB) and includes more severe channel impairments, making it a more challenging benchmark for evaluating model robustness and generalization.

The two datasets cover both analog and digital modulation schemes and incorporate various channel impairments, including multipath fading, frequency offset, sample-rate offset, and additive noise, thereby providing a comprehensive test of model robustness and generalization under varying noise levels and modulation complexities. This study adopts a consistent partitioning and preprocessing pipeline for both datasets. Following the principle of stratified sampling, we divide the data into training, validation, and test sets at a ratio of 8:1:1 for model training, validation, and final performance evaluation. Stratification is performed jointly over modulation class and SNR bin, so that each (class, SNR) group is proportionally represented in every split. The split is generated by a seeded random shuffle (see below), and the same partition is shared by all models within a given run to ensure a fair comparison.

#### 4.1.2. Data Preprocessing and Adaptation

Given the wide SNR range of the RML2016 datasets (−20 dB to +18 dB), the amplitude of the raw I/Q signals fluctuates dramatically. We adopt a two-stage normalization strategy to stabilize training: first, the mean and standard deviation required for Z-score normalization are estimated using only the training set, and the same statistics are applied to the training, validation, and test sets to preserve consistency and avoid information leakage; second, per-sample Z-score normalization (subtracting the sample mean and dividing by the sample standard deviation, computed independently over all channels and time steps) is applied to each input sample during the model’s forward pass to eliminate absolute power differences, forcing the model to focus on the relative structural features of the signal.

We note that per-sample normalization intentionally removes absolute amplitude information, which is correlated with SNR. This is a deliberate design choice: the goal of AMR is to identify the modulation type, not to estimate SNR, and the discriminative cues for modulation—constellation geometry, phase transitions, and spectral envelope shape—are encoded in the relative structure of the I/Q waveform rather than in its absolute power level. Without per-sample normalization, the model risks overfitting to SNR-dependent amplitude scales instead of learning modulation-specific patterns, particularly given the 38 dB SNR range spanned by the dataset. The first-stage Z-score normalization preserves relative amplitude differences across samples, while the second-stage per-sample normalization further removes residual channel-dependent gain variations. This design is also consistent with the prior deep-learning-based AMR/AMC literature, which treats signal preprocessing and input representation as important components of the recognition pipeline [1]. The comparative results reported below further suggest that removing absolute-power cues does not hinder modulation discrimination, since BioLAMR still achieves the highest accuracy in both low- and high-SNR regimes.

To bridge the modality gap between continuous signals and the pretrained transformer, we implement a dedicated data-adaptation pipeline that bypasses the standard discrete tokenizer. Specifically, the normalized I/Q sequences (2×128) are directly mapped into the continuous embedding space of GPT-2 through a convolutional projection layer. This transformation converts the raw signal into a sequence of high-dimensional vectors ($128\times d_{\mathrm{gpt}}$), preserving temporal continuity and phase information that would otherwise be lost through quantization, and thereby ensuring compatibility with the GPT-2 Small backbone interface.

#### 4.1.3. Implementation Details

All experiments were conducted on a high-performance workstation equipped with an NVIDIA RTX 4090 GPU (24GB VRAM) and an Intel Core i9-14900K CPU. We employed the AdamW optimizer with a weight decay of 0.01 to ensure stable convergence and effective regularization for the transformer-based architecture. To balance the rapid acquisition of task-specific features with the preservation of the pretrained model’s general sequence-modeling capability, we used a hierarchical learning-rate strategy. Specifically, a higher base learning rate of $5\times 10^{-4}$ was assigned to the randomly initialized modules (including the dual-domain feature extraction, embedding layer, and classification head) to facilitate fast adaptation. In contrast, a more conservative learning rate of $2.5\times 10^{-4}$ was applied to the unfrozen GPT-2 parameters (LayerNorm, position embeddings, attention blocks, and the MLP) to mitigate the risk of catastrophic forgetting. Learning rates were dynamically adjusted using the OneCycleLR scheduler, with a 30% warm-up phase to stabilize gradients during early training. A batch size of 128 was used to maintain reliable gradient estimates while maximizing memory efficiency. Training was capped at 50 epochs, with early stopping based on a patience of 5 epochs to prevent overfitting.

The model backbone was instantiated as GPT-2 Small (12 transformer blocks, 12 attention heads, 768 hidden units, approximately 124M parameters), chosen to balance long-range dependency modeling capability and training efficiency while maintaining acceptable inference cost on standard GPU hardware. For the lightweight dual-domain fusion module, the hyperparameters were determined empirically by grid search: the residual channel dimension was set to $d_{\mathrm{res}}=64$ to compress feature representations without introducing an information bottleneck; the convolutional kernel size was fixed at k=3 to capture local transient signal details; and the initial fusion weight was set to λ=0.1 to prioritize stable time-domain features during initialization. The classification head adopted a funnel-shaped projection design: 768→512→256→11 for dataset A and 768→512→256→10 for dataset B. A dropout rate of p=0.15 was used to improve the stability of feature refinement and enhance generalization under low-SNR conditions.

To assess result stability, every model was trained and evaluated over four independent runs with random seeds {42,128,256,512}. For each seed, the data partition was regenerated using the same stratified procedure described above, and the checkpoint with the highest validation accuracy was selected per run. Overall accuracy and F1 score in the comparison and main ablation tables are reported as mean ± standard deviation across these four runs. Statistical significance between BioLAMR and the strongest baseline was assessed via two-sided paired *t*-tests (α=0.05).

### 4.2. Comparison and Analysis

#### 4.2.1. Baseline Models

To comprehensively evaluate the performance of BioLAMR, we selected seven representative baselines spanning multiple paradigms, ranging from signal-to-image transformation methods to transformer-based sequence-modeling architectures. These baselines include MTF [64], CD [65], SigNet [66], ResNet+LSTM [67], AVGNet [68], CTGNet [69], and MCFormer [25]. Among them, MCFormer is a pure transformer encoder that directly models I/Q signal sequences and is therefore the most architecturally comparable baseline to BioLAMR. Collectively, these models examine signal characteristics from the perspectives of signal-to-image transformation, time-domain sequence modeling, spatiotemporal hybrid modeling, topological features, and pure transformer sequence modeling, representing diverse technical routes in the AMR literature. All baselines were re-implemented and retrained from scratch on the same data partitions, preprocessing pipeline, and hardware used for BioLAMR; no results were imported from the original publications. Each baseline was trained with the hyperparameters recommended in its respective paper (or, when not specified, determined by a small grid search on the validation set) to ensure both fairness and near-optimal performance.

#### 4.2.2. Performance Across SNR Regimes

Recognition accuracy as a function of signal-to-noise ratio is one of the most intuitive metrics for evaluating AMR model performance. Table 3 compares the performance of all models on two datasets (A: RML2016.10a, B: RML2016.10b).

**Figure 5:**
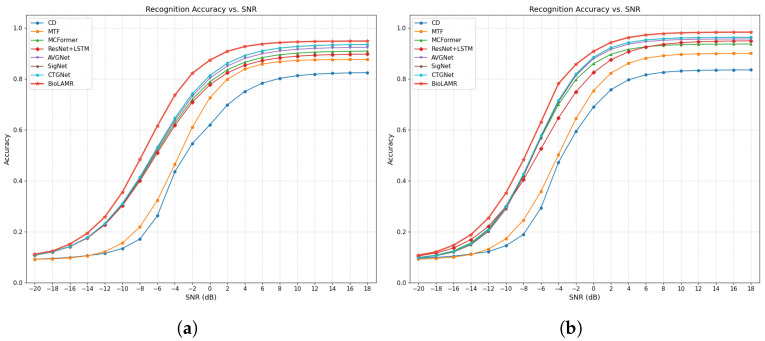
Recognition accuracy of eight methods as a function of SNR, with SNR ranging from −20 dB to +18 dB. (**a**) RML2016.10a; (**b**) RML2016.10b.

Figure 5 shows BioLAMR’s performance across different SNR regimes. In the high-SNR regime (0 dB to +18 dB), BioLAMR achieves 96.72% on dataset B and 93.21% on dataset A, both of which are the highest values in the table. Compared with MCFormer, the most architecturally comparable pure-transformer baseline, BioLAMR improves high-SNR accuracy by 5.26 and 5.19 percentage points on datasets A and B, respectively. This result suggests that the combination of dual-domain feature modeling and an LLM backbone enhances discrimination among high-order modulation types such as 16QAM and 64QAM: the time-domain branch preserves amplitude and phase details, whereas the frequency-domain branch supplements spectral morphology and energy-distribution information; meanwhile, the GPT-2 backbone integrates discriminative cues across time steps through global dependency modeling.

It is worth noting that although RadioML2016.10b includes stronger channel impairments, its overall accuracy is slightly higher than that of RadioML2016.10a for most models in our experiments. We believe this mainly reflects benchmark composition rather than genuinely easier channel conditions. First, RadioML2016.10b excludes AM-SSB and therefore contains 10 classes instead of 11, which reduces average class ambiguity. Second, RadioML2016.10b is substantially larger than RadioML2016.10a (1,200,000 versus 220,000 total samples), so under the same 8:1:1 stratified split it still provides proportionally more training data. This larger sample size can partially offset the stronger impairments by allowing more stable parameter estimation. Third, the two datasets differ in class composition and inter-class separability, so their mean accuracies should not be interpreted as a direct ranking of physical difficulty. Consequently, the two datasets are better regarded as complementary benchmarks than directly ranked by overall accuracy alone.

In the low-SNR regime (−20 dB to −2 dB), BioLAMR achieves 36.78% and 38.14% on datasets A and B, respectively, again the highest values in the table. Compared with MCFormer, BioLAMR improves low-SNR accuracy by 3.27 and 3.01 percentage points on datasets A and B, respectively. This result indicates that when noise is severe, the combination of an LLM backbone and dual-domain feature modeling still provides a measurable advantage. A plausible explanation is that when local instantaneous features are weakened by noise, the model can still exploit longer-range contextual dependencies and complementary time–frequency information to recover more stable discriminative cues. However, this observation is still based mainly on empirical performance, and the precise mechanism by which pretraining priors contribute to denoising and structure recovery warrants further study. Two-sided paired *t*-tests over four independent runs confirm that BioLAMR significantly outperforms every baseline on both datasets (all p<0.002, α=0.05). The tightest comparison is against CTGNet, the strongest baseline, where the gap remains significant (p=0.0013 for dataset A; p=0.0010 for dataset B).

#### 4.2.3. Confusion Matrix Analysis

**Figure 6:**
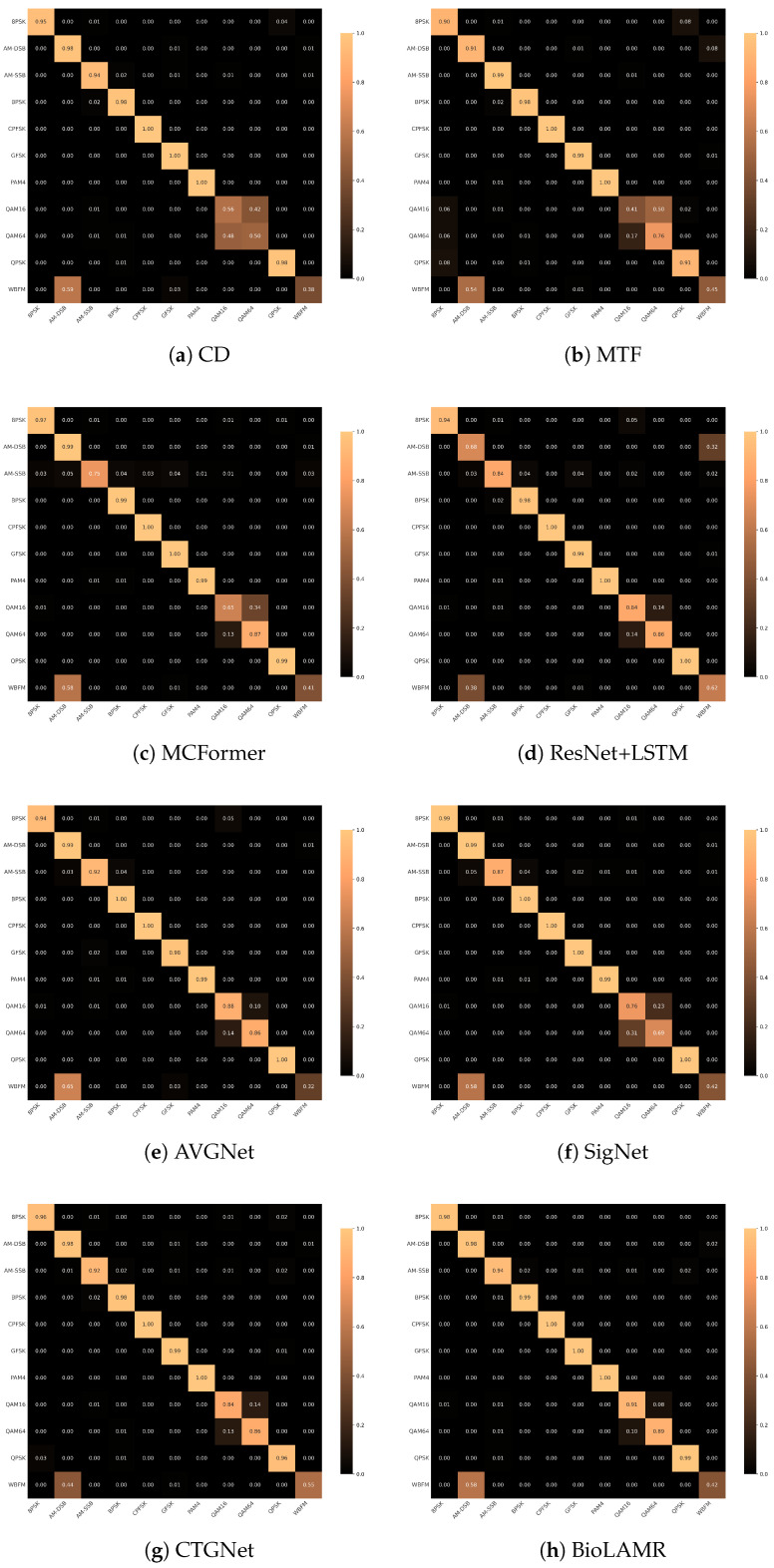
Confusion matrices of different models on the RML2016.10a dataset at SNR = 10 dB, including seven baselines and the proposed method.

To further analyze classification performance, Figure 6 presents the confusion matrix of BioLAMR on the RML2016.10a dataset at an SNR of 10 dB. We select 10 dB as a representative high-SNR operating point, at which random noise interference is largely suppressed. Under this condition, the remaining classification errors mainly arise from intrinsic structural similarities among modulation types, such as constellation overlap between 16QAM and 64QAM, rather than from channel noise, thereby providing a clearer view of the model’s fine-grained feature-extraction capability.

In Figure 6, a brighter diagonal indicates higher per-class classification accuracy. Compared with the baselines, BioLAMR exhibits a clearer diagonal pattern, suggesting more stable decision boundaries across multiple modulation classes. Notably, signal-to-image transformation methods such as MTF and CD perform relatively poorly on spectrally and structurally similar modulations because the 2D image representation loses part of the phase information, especially when distinguishing 16QAM from 64QAM. By contrast, BioLAMR reduces errors for these confusing classes, further indicating that the combination of dual-domain features and an LLM backbone is beneficial for fine-grained modulation discrimination.

#### 4.2.4. Feature Space Visualization

**Figure 7:**
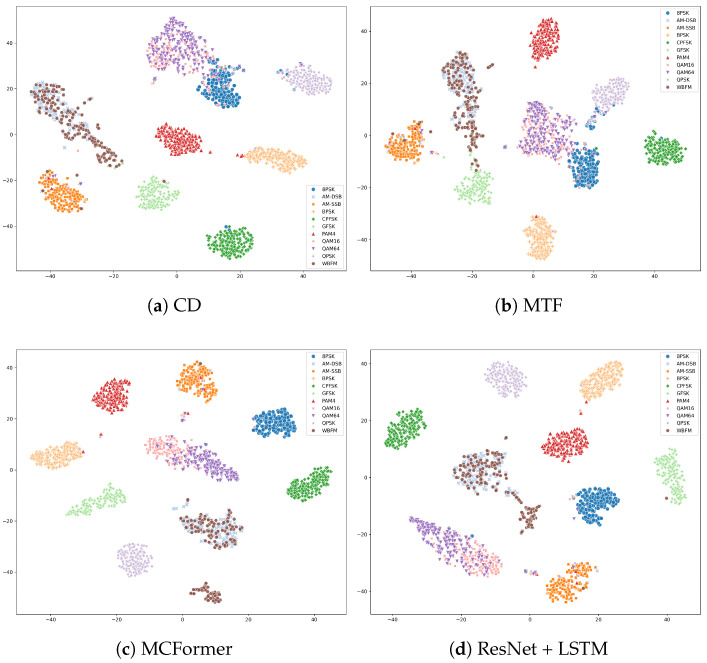

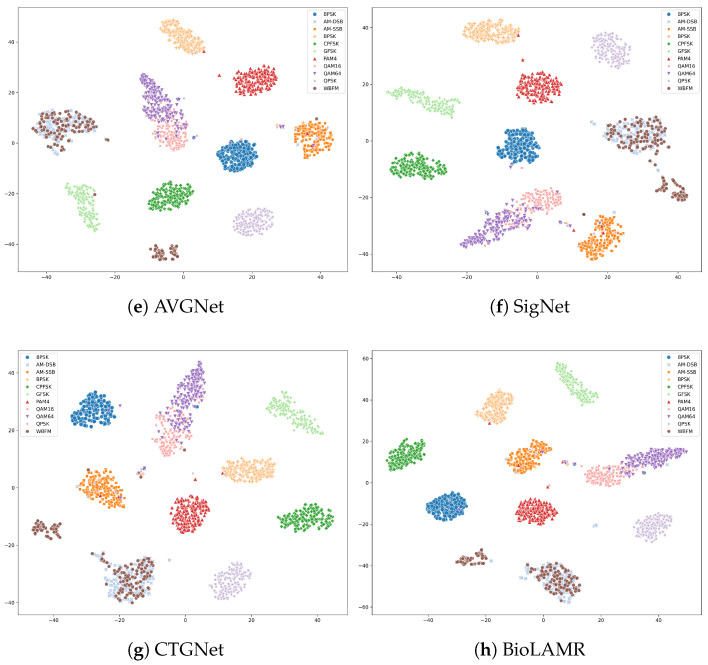
t-SNE visualization of learned feature representations for different models on the RML2016.10a dataset at SNR = 10 dB, including seven baselines and the proposed method.

Finally, to visualize the learned feature representations, we use t-SNE to project the high-dimensional features from the model’s final hidden layer into a two-dimensional space. For all models, t-SNE was run with perplexity = 30, 1000 iterations, learning rate = 200, and a fixed random seed of 42; 200 samples per modulation class were randomly drawn at SNR = 10 dB, yielding 2200 points in total. As shown in Figure 7 for the RadioML2016.10a dataset, the feature clusters produced by BioLAMR are more compact and better separated than those of the baseline models. We stress that t-SNE is a nonlinear stochastic projection and the resulting plots should be interpreted as qualitative, complementary evidence rather than a quantitative metric; in particular, inter-cluster distances in the embedding do not directly reflect distances in the original feature space. This visualization result indirectly supports the effectiveness of dual-domain feature extraction and pretrained transformer knowledge transfer, suggesting that the proposed method learns more discriminative feature representations.

#### 4.2.5. Computational Efficiency Analysis

To comprehensively assess the practical deployability of BioLAMR, Table 4 compares the computational efficiency of different models, including total parameters, trainable parameters, FLOPs, and single-sample GPU inference latency. All measurements were performed on the RadioML2016.10a dataset.

As shown in Table 4, BioLAMR exhibits a clear trade-off between parameter scale and inference cost. Training efficiency: owing to the hierarchical freezing strategy, the actual number of trainable parameters is only 11.1M (about 8.9% of the total), which is lower than that of large fully trainable baselines such as CD, MTF, and SigNet (each with about 23.5 M trainable parameters). This helps reduce training cost, memory demand, and the risk of overfitting. Inference efficiency: BioLAMR requires 3.2 ms for single-sample GPU inference, which is faster than the large fully trainable baselines (CD, MTF, and SigNet, 5.5–7.2 ms), although it remains slower than several lightweight baselines such as ResNet+LSTM, AVGNet, and CTGNet (0.45–0.65 ms). Notably, BioLAMR has the highest FLOPs (11.16 G) yet achieves lower latency than CD, MTF, and SigNet (∼4.1 G FLOPs each). This is because the transformer backbone consists almost entirely of dense matrix multiplications that saturate GPU parallelism, whereas image-based baselines involve signal-to-image conversion pipelines and irregularly structured convolutions that underutilize GPU throughput. Therefore, BioLAMR’s main advantage does not lie in absolute inference speed, but in its balance between higher recognition accuracy and acceptable inference cost. Accuracy–efficiency trade-off: for robotic communication or human–robot collaboration scenarios that require robust link sensing, this trade-off may be practically valuable, although system-level deployability must still be evaluated in conjunction with the target hardware platform, latency budget, and workload. A preliminary edge-device assessment is presented in Section 5.

### 4.3. Ablation Studies

To verify the contribution of each core component in the BioLAMR architecture, we conduct a series of ablation experiments under identical training configurations, focusing on changes in mean accuracy and low-SNR performance.

#### 4.3.1. Efficacy of Dual-Domain Feature Extraction and Fusion

We construct four model variants to assess the contributions of the time–frequency dual-domain structure and the fusion strategy:

- Time-Only: uses only the time-domain branch $X_{t}$, removing the frequency-domain branch and the fusion module.
- Freq-Only: uses only the frequency-domain branch $X_{f}$.
- Concat-Fusion: retains both branches but removes the LDDF attention mechanism; the features are directly concatenated and fused by a linear layer.
- LDDF (Proposed): the complete lightweight dual-domain attention fusion architecture.

As shown in Table 5, the four variants reveal a clear hierarchy. Using only the time-domain or frequency-domain branch yields 60.98%/60.06% on dataset A, confirming that neither domain alone captures sufficient discriminative information. Combining both branches via simple concatenation raises accuracy to 62.36%, but the proposed LDDF fusion further improves it to 64.99%—a ∼2.6 pp gain that demonstrates the value of attention-driven adaptive weighting over naive feature merging. From a biomimetic perspective, these results provide functional grounding for the two biologically motivated design choices embedded in the LDDF module. First, the complementarity between the time and frequency branches mirrors the cochlea’s simultaneous frequency decomposition and temporal-transient preservation: modulated signals inherently carry distinct information in each domain (e.g., symbol transitions vs. spectral envelopes), and the ∼4 pp gap between single-branch and dual-branch variants confirms that the parallel-then-fuse design captures genuinely complementary cues. Second, the channel and spatial attention paths correspond, respectively, to feature-based (what) and position-based (where) selective attention identified in cognitive neuroscience [33]; the ∼2.6 pp improvement over Concat-Fusion validates that this specific dual-path combination adds value beyond unstructured feature merging.

#### 4.3.2. Signal Embedding and Modality Adaptation

To validate the effectiveness of the convolutional embedding mechanism in mapping signals to the backbone feature space, we compare three embedding strategies:

- Native LLM: quantizes continuous I/Q values into discrete integers and directly applies GPT-2’s native vocabulary embedding, simulating the naive “signal-as-language” assumption.
- Linear Projection: uses a single-layer linear transformation to independently map each signal sampling point to GPT-2’s embedding space without introducing temporal context.
- Conv Embedding (Proposed): uses a 1D convolutional layer to locally aggregate adjacent sampling points before projection, preserving signal continuity and phase correlation.

As shown in Table 6, the experiments on the RadioML2016.10a dataset highlight the importance of the embedding strategy. The Native LLM method fails to converge effectively, with accuracy remaining close to random guessing, indicating that naively discretizing the signal into tokens is ineffective in the current setting. Quantization maps continuous amplitude and phase changes to discrete symbols and destroys key information such as phase differences, making it difficult for GPT-2 to reconstruct the physical structure of the signal from vocabulary embeddings.

The linear projection approach provides a clear improvement, reaching an average accuracy of 57.38%, which confirms that continuous representations are necessary. However, its performance bottleneck remains evident: because it lacks a local receptive field, the model cannot effectively capture phase continuity between adjacent sampling points and remains insufficiently sensitive to subtle constellation differences in high-order modulations.

By contrast, the proposed Conv Embedding strategy performs best, reaching an average accuracy of 64.99%, which is 7.61 percentage points higher than that of linear projection. Convolutional embedding introduces a local inductive bias through a receptive field of K=3, allowing each embedding vector to integrate phase trends from adjacent time steps and endowing the input sequence with preliminary smoothness and contextual semantics. This design reduces the burden on the backbone when modeling low-level waveform correlations, allowing more modeling capacity to be allocated to high-level modulation-pattern recognition.

#### 4.3.3. Hierarchical Parameter Fine-Tuning Strategy and Pretraining Efficacy

A key component of BioLAMR is the hierarchical parameter fine-tuning strategy inspired by hierarchical cortical processing [70]. This strategy selectively unfreezes a subset of GPT-2 parameters (LayerNorm, position embeddings, attention blocks, and MLP layers) and assigns different learning rates to pretrained layers and newly introduced modules, thereby balancing knowledge preservation and task adaptation. To evaluate this design more systematically, and to disentangle the effects of the *transformer architecture itself* from those of GPT-2 pretrained knowledge transfer, we compare the following six variants:

- Full Fine-tuning: load pretrained weights, unfreeze all parameters, and optimize the entire model with a uniform learning rate of $5\times 10^{-4}$.
- Full Freeze: load pretrained weights, freeze all GPT-2 parameters, and train only the dual-domain fusion module, convolutional embedding layer, and classifier head.
- Uniform LR: load pretrained weights and adopt the same unfreezing pattern as the proposed method, but optimize all trainable parameters with a uniform learning rate of $5\times 10^{-4}$.
- Training from Scratch: randomly initialize the GPT-2 backbone with Xavier uniform initialization, train all parameters, and use a uniform learning rate of $5\times 10^{-4}$ to isolate the pure architectural contribution.
- Hierarchical Unfreezing from Scratch: apply random initialization together with the same hierarchical unfreezing and learning-rate schedule as the proposed method to test whether the strategy remains effective without pretrained knowledge.
- Hierarchical Fine-tuning (Proposed): load pretrained weights and apply selective unfreezing with hierarchical learning rates ($5\times 10^{-4}$ for newly added modules and $2.5\times 10^{-4}$ for pretrained layers).

As shown in Table 7, the six variants exhibit a clear performance ordering, which helps isolate the respective effects of parameter-update strategy and pretrained knowledge transfer.

Limitation of full freezing: when all GPT-2 parameters remain frozen, the model reaches only 56.15% and 58.47% on datasets A and B, respectively, which is the weakest result among the pretrained variants. Notably, this result is even lower than that of the linear-projection-embedding variant with hierarchical fine-tuning (57.38%, see Table 6), indicating that a stronger convolutional embedding alone cannot compensate for a fully frozen GPT-2. The mismatch between pretrained text features and wireless signal characteristics still requires moderate parameter adaptation to reduce cross-modal discrepancy. This limitation is especially apparent in the low-SNR regime (27.82%/29.16%).

Limitation of full fine-tuning: unfreezing all parameters with a uniform learning rate improves performance to 61.52% and 63.87%, but it still remains inferior to the proposed method. A plausible explanation is that updating approximately 125 million parameters at a relatively aggressive step size on a limited dataset increases the risk of catastrophic forgetting, thereby weakening the long-range dependency priors acquired during pretraining. This result suggests that, under the current data scale and optimization setting, retaining part of the pretrained representation is more beneficial than fully rewriting it.

Key role of hierarchical learning rates: the Uniform LR variant (63.14%/65.62%) already outperforms the previous two, showing that selective unfreezing itself is effective. However, the proposed hierarchical fine-tuning strategy further improves accuracy to 64.99%/67.43%, surpassing Uniform LR by 1.85 and 1.81 percentage points on datasets A and B, respectively. The difference is particularly pronounced in the low-SNR regime (1.86 and 1.96 percentage points), indicating that applying more conservative update steps to pretrained layers helps preserve GPT-2’s long-sequence modeling priors and maintain more stable performance under strong noise. Overall, compared with Full Freeze and Full Fine-tuning, hierarchical fine-tuning improves overall accuracy by 8.84%/8.96% and 3.47%/3.56%, respectively, demonstrating a better balance between knowledge preservation and task adaptation.

Transfer performance gain from pretrained knowledge: comparing Training from Scratch (57.49%/59.84%) with the proposed method (64.99%/67.43%) shows that pretrained GPT-2 brings an overall accuracy gain of 7.50 and 7.59 percentage points. This gain is especially evident in the low-SNR regime, where the margin reaches 6.92 percentage points on both datasets. This suggests that the long-range sequence-dependency modeling capability learned by GPT-2 from large-scale text corpora can be effectively transferred to the signal domain and help recover useful structural information from noise-corrupted sequences. Notably, the from-scratch model already outperforms some lightweight baselines, indicating that the transformer architecture itself has meaningful sequence-modeling advantages; however, the roughly 7.5-point gap relative to the proposed method shows that architectural advantage alone cannot fully explain BioLAMR’s performance gain, and pretrained knowledge transfer plays a crucial role. Conditional effectiveness of the hierarchical strategy: comparing the two from-scratch variants shows that hierarchical unfreezing reduces performance without pretraining by 6.05 and 6.06 percentage points. This reveals a key prerequisite: the frozen parameters must encode meaningful prior knowledge. If the frozen layers contain only random weights, the lower transformer blocks output unstructured features, and the trainable upper layers cannot build effective representations on that basis. In contrast, under pretrained initialization, the frozen lower layers preserve generic sequence-modeling capability and provide a stable foundation for upper-layer task-specific adaptation. This asymmetry also provides the strongest functional grounding for the cortical-hierarchy analogy [34]: just as hierarchical processing in the auditory cortex depends on generic representations already established in primary areas, the hierarchical fine-tuning strategy is effective only when the frozen layers carry meaningful pretrained priors—an outcome that would not be expected from an arbitrary engineering heuristic.

### 4.4. Summary

Through systematic comparative experiments and ablation studies, this section validates the effectiveness of BioLAMR from multiple perspectives. BioLAMR outperforms all seven baselines on both benchmarks, and confusion-matrix and t-SNE analyses further confirm its fine-grained discriminative capability. The three ablation studies isolate the contributions of dual-domain fusion, convolutional embedding, and hierarchical fine-tuning, showing that each component is individually necessary and that their combination yields the best overall performance. The pretraining-efficacy analysis reveals that pretrained knowledge transfer accounts for roughly 7.5 pp of accuracy gain beyond pure architectural advantage, and that the hierarchical strategy is effective only when meaningful pretrained priors are present. The computational-efficiency analysis shows that BioLAMR achieves a reasonable trade-off between recognition accuracy and inference cost despite its large total parameter count. Importantly, each biologically inspired module is independently validated by controlled ablation experiments that provide functional—not merely metaphorical—grounding for the design choices (see the biomimetic discussions in Section 4.3.1 and Section 4.3.3).

## 5. Discussion: Future Directions for Robotic and Human–Robot Collaborative Communications

The current validation of BioLAMR is limited to public benchmark datasets (RadioML2016.10a and RadioML2016.10b) and has not yet involved system-level deployment or closed-loop testing on real robotic platforms or embedded hardware. Therefore, the discussion in this section should be understood as outlining potential future directions that indicate plausible application paths for the method in robotic and human–robot collaborative communication scenarios, rather than as presenting already verified system-level conclusions. From the perspective of Biomimetics, the value of BioLAMR lies in providing a biomimetically inspired communication-sensing algorithm prototype: the dual-domain parallel feature extraction is inspired by multipath analysis in biological perception, whereas hierarchical fine-tuning is motivated by the idea of preserving generic representations and gradually adapting them to new tasks. The following subsections discuss feasible pathways for future integration and validation across different application settings.

As with all studies evaluated on synthetic benchmarks, it should be noted that the RadioML2016 datasets are generated by software-defined radio simulation, and samples within the same (modulation class, SNR) group share underlying generation parameters. This introduces inter-sample correlation that is typically higher than in real over-the-air captures—a well-known characteristic of these widely used benchmarks. Our (class, SNR)-stratified splitting mitigates partition-level bias by ensuring proportional representation, but residual statistical dependence across partitions is inherent to the dataset design. Accordingly, the accuracy figures reported herein are best viewed as benchmark-level performance indicators; generalisation to field conditions will require further validation on real captured signals, as noted above.

Regarding the biomimetic design philosophy, the biological analogies used in this work—auditory time–frequency decomposition, selective attention, and cortical hierarchical processing—serve as design-level inspirations rather than mechanistic reproductions of biological systems. We use the term “biomimetically inspired” throughout to reflect this distinction. Importantly, the ablation studies in Section 4.3.1 and Section 4.3.3 provide functional, not merely metaphorical, grounding for each biologically motivated module: every bio-inspired component is independently validated by controlled experiments that demonstrate its contribution beyond surface-level analogy.

### 5.1. Algorithm Positioning and Mapping to Scenario Demands

The positioning of this study is as follows: it is an algorithm-level investigation motivated by the need for robust wireless-link sensing in robotic and human–robot collaborative communication scenarios, validated on public benchmark datasets, and providing a reusable algorithm prototype for future system-level integration on robotic platforms. This positioning differs from that of an end-to-end solution that has already been deployed and verified in a robotic system. The relationship between the two is analogous to the distinction, in the Biomimetics field, between proposing a biomimetic sensing-mechanism prototype and integrating that prototype into a complete robotic system.

To make the relationship between algorithm performance and scenario demands more explicit, Table 8 maps representative engineering requirements in robotic communications to the key design characteristics and experimental results of BioLAMR, thereby illustrating the scenario-adaptation potential of the proposed method.

It should be emphasized that the above mapping is based on algorithm-level conclusions derived from standard benchmark datasets. Moving from algorithm validation to actual robotic-system deployment still requires further evaluation using real collected signals, hardware constraints, and closed-loop control scenarios.

To the best of our knowledge, there is currently no publicly available AMR benchmark specifically designed for robotic communication scenarios. Constructing such a dataset—incorporating realistic industrial multipath, Doppler effects from mobile platforms, and protocol-coexistence interference—is itself a valuable direction for future work and would enable more targeted validation of methods like BioLAMR.

### 5.2. Industrial Collaborative Robots and Human–Robot Collaboration

In intelligent manufacturing and Industry 4.0 environments, collaborative robots (cobots) share workspaces with human operators and need to exchange control commands and sensor data in real time through multiple wireless protocols, such as Wi-Fi, 5G NR, and wireless extensions of industrial Ethernet [10]. The electromagnetic environment in factory workshops is typically highly complex, with severe co-channel interference and multipath reflection. In such scenarios, BioLAMR is more appropriately viewed as a front-end modulation-sensing module on the receiver side, supporting link selection, demodulation configuration, or interference monitoring under protocol coexistence and channel disturbance. For collaborative robotic systems, the value of such a module lies in providing more stable low-level signal understanding for upper-level control and safety communications rather than replacing the entire communication protocol stack. Practical deployment will still require validation with site-collected data, edge-hardware constraints, and system-latency budgets.

### 5.3. UAV Swarms and Teleoperation Scenarios

In cooperative unmanned aerial vehicle (UAV) missions, reliable communication links must be maintained both among UAVs and between UAVs and ground control stations [12]. During flight, UAVs face rapidly changing channel conditions, including Doppler shift and shadow fading, while spectrum resources become especially constrained in multi-UAV coordination. BioLAMR may serve as a cognitive-radio front-end sensing unit that provides input for dynamic spectrum access, link reconfiguration, and abnormal-link diagnosis. In teleoperation scenarios [53], modulation recognition alone cannot resolve delay and immersion issues, but it may improve the reliability of link sensing in complex electromagnetic environments and thereby support higher-level fault-tolerant control and adaptive communication strategies.

### 5.4. Wearable Interactive Devices and Brain–Computer Interfaces

Emerging wearable intelligent devices and brain–computer interface (BCI) systems typically rely on low-power wireless protocols such as BLE and Zigbee to transmit physiological data [15,32,50,54]. Because these devices operate in ISM bands, they are subject to severe interference from other devices. The design of BioLAMR, inspired by auditory parallel analysis and selective attention, may provide a useful reference for communication sensing under weak-signal conditions. For the Biomimetics community, its significance lies in demonstrating how bio-inspired multipath analysis and hierarchical adaptation mechanisms can be transferred to wireless-signal understanding problems and support communication scenarios involving interactive devices.

### 5.5. From Biomimetically Inspired Communication Sensing to Robotic Multimodal Systems

From a broader robotics perspective, BioLAMR can be regarded as a prototype of “biomimetically inspired communication sensing”: it combines biologically inspired dual-domain analysis with a large language model and thereby provides an interface for the future fusion of wireless signals with other modalities such as vision and touch.

As a preliminary edge-device assessment, we profiled BioLAMR on an NVIDIA Jetson AGX Orin: the inference latency was 24 ms and the peak memory usage was 937 MB, providing initial evidence that near-real-time operation on embedded platforms is achievable. A more comprehensive edge evaluation—including ONNX/TensorRT optimization, multi-model comparison on edge hardware, and power-consumption analysis—is left to future work.

Future work can proceed along three directions: (1) closed-loop validation on real robotic or human–robot collaboration platforms to evaluate the actual contribution of the method to link adaptation and task stability; (2) extending the framework to broader wireless-sensing tasks such as signal detection, protocol recognition, and interference analysis; and (3) improving deployability on edge robotic platforms by combining lightweight fine-tuning, distillation, and quantization techniques.

### 5.6. Summary of Limitations

For transparency and to facilitate future improvements, Table 9 consolidates the main limitations of the current study together with potential mitigation directions.

A related methodological consideration is that BioLAMR currently treats I/Q components as two real-valued channels in order to remain compatible with the real-valued pretrained GPT-2 Small backbone. As demonstrated by Lei et al. [47], complex-domain processing is a viable alternative for AMR and could in principle be introduced into BioLAMR’s front-end modules to better preserve real–imaginary coupling. A promising future direction is therefore a hybrid design that combines complex-valued front-end processing with a complex-to-real interface before the pretrained transformer.

A further consideration is that the current anti-forgetting mechanism relies on hierarchical unfreezing and conservative learning rates, which are effective for the fixed downstream AMR task studied here but may not suffice for continual or multi-environment adaptation. Recent work has shown that projecting parameter updates into the null space of a low-rank feature covariance matrix can effectively mitigate forgetting in dynamic wireless adaptation [71]. Because such a mechanism constrains updates to directions that preserve the network outputs on previously learned data, it is potentially applicable to BioLAMR as well. However, the cited method is mainly developed for phase-wise adaptation in dynamic environments and further introduces selective forgetting when model capacity becomes saturated, which goes beyond the scope of the present single-task study. Incorporating feature-covariance-based null-space fine-tuning into BioLAMR for continual or multi-environment AMR adaptation therefore remains a promising future direction.

## 6. Conclusions

Motivated by the prospective need for robust wireless sensing in robotic and human–robot collaborative systems, this study proposes BioLAMR, a biomimetically inspired large language model adaptation framework for automatic modulation recognition. The framework combines three elements: a lightweight dual-domain fusion module inspired by the auditory system, a convolutional embedding mechanism for continuous I/Q signals, and a hierarchical parameter fine-tuning strategy inspired by cortical hierarchical processing. Together, these components transfer the sequence-modeling capability of GPT-2 Small to the AMR task. On the public RadioML2016.10a and RadioML2016.10b benchmarks, BioLAMR achieves overall accuracies of 64.99% and 67.43%, respectively, outperforming seven comparison baselines.

The main significance of this study is that it provides an algorithm-level prototype for robotic communication sensing from a Biomimetics perspective. Methodologically, it shows how biologically inspired parallel time–frequency analysis and hierarchical adaptation can be applied to wireless-signal recognition. At the application level, robotic and human–robot collaborative communication remains the motivation rather than a validated deployment. The present conclusions are based entirely on public benchmark datasets, and validation with real over-the-air signals, system-level robotic deployment, and broader edge-hardware evaluation remain future work.

In summary, BioLAMR offers a feasible way to combine biomimetically inspired perception with pretrained transformer modeling for wireless-signal understanding. It also provides a concrete starting point for future communication-sensing modules in robotic and human–robot interaction systems. 

## Figures and Tables

**Table 1:** Mathematical notation for dual-domain feature extraction and fusion.

Symbol	Dimension	Description
*x*	$R^{C\times L}$	Input I/Q signal (raw)
$x_{\mathrm{norm}}$	$R^{C\times L}$	Normalized input signal
$X_{t}$	$R^{C\times L}$	Time-domain features
$X_{f}$	$R^{C\times L}$	Frequency-domain features
$X_{c}$	$R^{2C\times L}$	Concatenated features
$M_{c}$	$R^{2C\times 1}$	Channel attention map
$M_{s}$	$R^{1\times L}$	Spatial attention map
*Y*	$R^{C\times L}$	Fused output features
λ	R	Learnable residual weight
σ:R→(0,1)	–	Sigmoid activation function
⊗	–	Element-wise multiplication (Hadamard product)
∘	–	Function composition
*C*	N	Number of channels (I/Q dual channels, C=2)
*L*	N	Sequence length (e.g., L=128)
*B*	N	Batch size

**Table 2:** Hierarchical parameter-unfreezing scheme for GPT-2 Small (12 blocks, $d_{\mathrm{gpt}}=768$). Layer indices 0–11 are ordered from bottom to top.

Component	Layers	Parameters	Trainable
Token embedding ($V\times d_{\mathrm{gpt}}$)	–	38.6 M	Frozen
Position embedding ($L_{\max}\times d_{\mathrm{gpt}}$)	–	0.8 M	Yes
LayerNorm (γ,β)	Blocks 0–11 + final	38.4 K	Yes
Self-Attention (Q/K/V/O)	Blocks 0–9	23.6 M	Frozen
Self-Attention (Q/K/V/O)	Blocks 10–11	4.7 M	Yes
FFN (MLP)	Blocks 0–10	51.9 M	Frozen
FFN (MLP)	Block 11	4.7 M	Yes
Dual-domain fusion + embedding + classifier	–	0.9 M	Yes
Trainable/Total		11.1 M/125.2 M (8.9%)	

**Table 3:** Performance comparison (Overall Acc. and F1 reported as mean ± std over four runs; Dataset A: RML2016.10a, B: RML2016.10b). Bold indicates the best result.

Model	Dataset	Acc. vs. SNR (%)		Overall Acc. (%)	Recall	F1 Score
		−20 — −2	0 — 18			
CD [65]	A	21.50	77.50	49.50 ± 0.52	0.4950	0.5085 ± 0.0081
	B	22.50	80.50	51.50 ± 0.45	0.5150	0.5278 ± 0.0070
MTF [64]	A	22.80	84.60	53.70 ± 0.47	0.5370	0.5519 ± 0.0072
	B	24.50	87.00	55.75 ± 0.43	0.5575	0.5735 ± 0.0063
ResNet+LSTM [67]	A	33.05	86.76	59.91 ± 0.39	0.5991	0.6141 ± 0.0041
	B	34.38	92.16	63.27 ± 0.36	0.6338	0.6517 ± 0.0035
MCFormer [25]	A	33.51	87.95	60.73 ± 0.42	0.6073	0.6224 ± 0.0057
	B	35.13	91.53	63.33 ± 0.37	0.6333	0.6490 ± 0.0052
AVGNet [68]	A	33.87	89.43	61.65 ± 0.35	0.6172	0.6345 ± 0.0034
	B	34.94	94.03	64.49 ± 0.30	0.6483	0.6669 ± 0.0031
SigNet [66]	A	34.18	90.52	62.35 ± 0.33	0.6235	0.6382 ± 0.0043
	B	34.76	94.66	64.71 ± 0.28	0.6471	0.6655 ± 0.0035
CTGNet [69]	A	34.24	90.53	62.39 ± 0.32	0.6239	0.6389 ± 0.0029
	B	35.25	94.68	64.96 ± 0.28	0.6497	0.6746 ± 0.0026
**BioLAMR (Proposed)**	A	**36.78**	**93.21**	**64.99 ± 0.31**	**0.6515**	**0.6701 ± 0.0032**
	B	**38.14**	**96.72**	**67.43 ± 0.27**	**0.6758**	**0.6993 ± 0.0025**

**Table 4:** Computational efficiency comparison of different models (measured on RadioML2016.10a).

Model	Total Params.	Train. Params.	FLOPs (G)	Latency (ms)
CD [65]	23.5 M	23.5 M	4.08	5.8
MTF [64]	23.5 M	23.5 M	4.11	7.2
ResNet + LSTM [67]	2.1 M	2.1 M	0.34	0.45
MCFormer [25]	0.4 M	0.4 M	0.05	0.78
AVGNet [68]	2.8 M	2.8 M	0.45	0.52
SigNet [66]	23.5 M	23.5 M	4.07	5.5
CTGNet [69]	3.5 M	3.5 M	0.58	0.65
BioLAMR	125.2 M	11.1 M	11.16	3.2

**Table 5:** Ablation results for dual-domain fusion (Overall Acc. and F1 reported as mean ± std over four runs).

Model	Dataset	Acc. vs. SNR		Overall Acc.	Recall	F1 Score
		−20 — −2	0 — 18			
Time-Only	A	34.12%	87.83%	60.98 ± 0.52%	0.6098	0.6248 ± 0.0058
	B	35.46%	91.58%	63.52 ± 0.47%	0.6352	0.6502 ± 0.0049
Freq-Only	A	33.38%	86.73%	60.06 ± 0.57%	0.6006	0.6168 ± 0.0068
	B	34.22%	90.15%	62.19 ± 0.51%	0.6219	0.6365 ± 0.0059
Concat-Fusion	A	34.77%	89.94%	62.36 ± 0.44%	0.6236	0.6416 ± 0.0037
	B	35.85%	93.47%	64.66 ± 0.41%	0.6466	0.6638 ± 0.0033
LDDF (Proposed)	A	36.78%	93.21%	64.99 ± 0.31%	0.6515	0.6701 ± 0.0032
	B	38.14%	96.72%	67.43 ± 0.27%	0.6758	0.6993 ± 0.0025

**Table 6:** Ablation study of different signal embedding strategies on the RadioML2016.10a dataset.

Strategy	Mechanism	Avg. Acc.
Native LLM	Quantization + Vocab Lookup	∼9.09% (Failure)
Linear Projection	Point-wise Linear Transform	57.38%
Conv Embedding (Proposed)	Conv1d + Aggregation	64.99%

**Table 7:** Comprehensive ablation results for hierarchical parameter fine-tuning and pretraining efficacy (Overall Acc. and F1 reported as mean ± std over four runs).

Strategy	Dataset	Acc. vs. SNR		Overall Acc.	Recall	F1 Score
		−20 — −2	0 — 18			
Random Initialization						
Training from Scratch	A	29.86%	85.12%	57.49 ± 0.62%	0.5749	0.5892 ± 0.0075
	B	31.22%	88.46%	59.84 ± 0.55%	0.5984	0.6138 ± 0.0064
Hierarchical Unfreezing from Scratch	A	24.56%	78.32%	51.44 ± 0.74%	0.5144	0.5278 ± 0.0092
	B	25.88%	81.68%	53.78 ± 0.70%	0.5378	0.5516 ± 0.0083
Pretrained Initialization						
Full Freeze	A	27.82%	84.48%	56.15 ± 0.66%	0.5615	0.5758 ± 0.0078
	B	29.16%	87.78%	58.47 ± 0.60%	0.5847	0.5996 ± 0.0067
Full Fine-tuning	A	32.45%	90.59%	61.52 ± 0.46%	0.6152	0.6298 ± 0.0040
	B	33.58%	94.16%	63.87 ± 0.44%	0.6387	0.6542 ± 0.0036
Uniform LR	A	34.92%	91.36%	63.14 ± 0.38%	0.6314	0.6478 ± 0.0031
	B	36.18%	95.06%	65.62 ± 0.35%	0.6562	0.6738 ± 0.0028
Hierarchical Fine-tuning (Proposed)	A	36.78%	93.21%	64.99 ± 0.31%	0.6515	0.6701 ± 0.0032
	B	38.14%	96.72%	67.43 ± 0.27%	0.6758	0.6993 ± 0.0025

**Table 8:** Mapping between representative robotic-communication requirements and BioLAMR’s algorithmic characteristics.

Robotic Communication Requirement	Corresponding AMR Requirement	BioLAMR Characteristic and Supporting Result
Strong interference and multipath fading in factory workshops	Robust recognition under low SNR	Accuracy of 36.78%/38.14% in the low-SNR regime (−20∼−2 dB), outperforming all comparison baselines
Coexistence of multiple protocols such as Wi-Fi, BLE, Zigbee, and 5G NR	Multiclass recognition covering mainstream modulation types	Supports 11/10 modulation classes and covers both digital and analog modulation schemes
Limited computing resources on edge terminals	Low inference cost and parameter efficiency	Only 11.1 M trainable parameters (8.9% of the total) and 3.2 ms GPU single-sample inference time
Real-time link sensing and front-end adaptation	End-to-end differentiability without handcrafted features	End-to-end training with automatically learned dual-domain features and no need for manually designed modulation descriptors
Discrimination of high-order modulations in complex electromagnetic environments	Fine-grained class separability	Dual-domain fusion plus the pretrained transformer backbone yields better confusion-matrix performance on 16QAM/64QAM and tighter t-SNE feature clusters

**Table 9:** Summary of current limitations and potential mitigation directions.

No.	Limitation	Impact	Mitigation Direction
L1	Validation limited to synthetic benchmarks (RadioML2016.10a/10b)	Generalization to real over-the-air signals is unverified	Collect and evaluate on real captured signal datasets
L2	No system-level deployment or closed-loop robotic integration	Practical applicability in robotic and human–robot collaborative scenarios remains prospective	Conduct on-platform integration and task-level evaluation
L3	The current study focuses on AMR only and does not examine transfer to broader wireless-sensing tasks	Task-level transferability of the framework remains open	Extend the framework to related tasks such as spectrum sensing or protocol recognition

## Data Availability

The datasets used in this study, including RadioML2016.10a and RadioML2016.10b, are publicly available benchmark datasets provided by DeepSig Inc. The complete training and inference source code for the proposed BioLAMR framework is publicly available at https://github.com/saber778899/BioLAMR (accessed on 17 April 2026). The repository contains the model definition, training scripts for both datasets, and environment configuration files sufficient to reproduce the main experimental results reported in this paper.
